# Effects of Sarcopenic Obesity and Its Confounders on Knee Range of Motion Outcome after Total Knee Replacement in Older Adults with Knee Osteoarthritis: A Retrospective Study

**DOI:** 10.3390/nu13113817

**Published:** 2021-10-27

**Authors:** Chun-De Liao, Shih-Wei Huang, Yu-Yun Huang, Che-Li Lin

**Affiliations:** 1 Master Program in Long-Term Care, College of Nursing, Taipei Medical University, Taipei 110301, Taiwan; 08415@s.tmu.edu.tw; 2 Department of Hysical Medicine and Rehabilitation, Shuang Ho Hospital, Taipei Medical University, New Taipei City 235041, Taiwan; 13001@s.tmu.edu.tw; 3 Department of Physical Medicine and Rehabilitation, School of Medicine, College of Medicine, Taipei Medical University, Taipei 11031, Taiwan; 4 Department of Pediatrics, New York University Langone Medical Center, New York, NY 10016, USA; huang-yu-yun@hotmail.com; 5 Department of Orthopedic Surgery, Shuang Ho Hospital, Taipei Medical University, New Taipei City 23561, Taiwan; 6 Department of Orthopedics, School of Medicine, College of Medicine, Taipei Medical University, Taipei 11031, Taiwan

**Keywords:** sarcopenic obesity, malnutrition, osteoarthritis, total knee replacement, range of motion

## Abstract

Sarcopenic obesity is closely associated with knee osteoarthritis (KOA) and has high risk of total knee replacement (TKR). In addition, poor nutrition status may lead to sarcopenia and physical frailty in KOA and is negatively associated with surgery outcome after TKR. This study investigated the effects of sarcopenic obesity and its confounding factors on recovery in range of motion (ROM) after total knee replacement (TKR) in older adults with KOA. A total of 587 older adults, aged ≥60 years, who had a diagnosis of KOA and underwent TKR, were enrolled in this retrospective cohort study. Sarcopenia and obesity were defined based on cutoff values of appendicular mass index and body mass index for Asian people. Based on the sarcopenia and obesity definitions, patients were classified into three body-composition groups before TKR: sarcopenic-obese, obese, and non-obese. All patients were asked to attend postoperative outpatient follow-up admissions. Knee flexion ROM was measured before and after surgery. A ROM cutoff of 125 degrees was used to identify poor recovery post-surgery. Kaplan-Meier curve analysis was performed to measure the probability of poor ROM recovery among study groups. Cox multivariate regression models were established to calculate the hazard ratios (HRs) of postoperative poor ROM recovery, using potential confounding factors including age, sex, comorbidity, risk of malnutrition, preoperative ROM, and outpatient follow-up duration as covariates. Analyses results showed that patients in the obese and sarcopenic-obese groups had a higher probability of poor ROM recovery compared to the non-obese group (all *p* < 0.001). Among all body-composition groups, the sarcopenic-obese group yielded the highest risk of postoperative physical difficulty (adjusted HR = 1.63, *p* = 0.03), independent to the potential confounding factors. Sarcopenic obesity is likely at the high risk of poor ROM outcome following TKR in older individuals with KOA.

## 1. Introduction

Obesity has become epidemic worldwide in populations with knee osteoarthritis (KOA) and has growing impacts for those who are undergoing total knee replacement (TKR) [1]. Due to this, overweight or obesity is strongly associated with disease progression of KOA [2,3], obesity has an increased risk of TKR [4,5], and the prevalence of obesity in patients undergoing TKR surgery is expected to increase [6]. In addition, obesity is a predominant risk factor for post-surgery complications and may affect short-term or long-term outcomes after TKR [7,8], among which the recovery in knee flexion range of motion (ROM) is of particular interest [9,10,11,12,13]. However, the effects of obesity on surgery outcomes, including ROM, regain after TKR remains conflict [1]. A number of previous studies have indicated that obesity negatively affects ROM recovery following TKR [7,14], whereas others had inconsistent conclusions [15]. Due to the fact that the rate of TKR grows rapidly in obese older individuals with KOA [16,17] and that the postoperative ROM gains are significantly associated with function recovery [12,18,19,20,21], there is an urgent need to further identify the effect of obesity on ROM recovery following TKR.

In addition to obesity, low muscle mass (i.e., sarcopenia) has been given as a risk factor for KOA and its disease progression [22,23,24]. Notably, the obese older individuals with KOA who are classified as having sarcopenia (i.e., sarcopenic obesity) may suffer potential risks of physical decline and disability [25,26,27]. Sarcopenic obesity is a condition referring to the coexistence of sarcopenia and obesity and threatens physical health in elderly populations [28], with an overall global prevalence of 11% in older adults [29]. Sarcopenic obesity has been identified to be closely associated with chronic diseases such as knee osteoarthritis (KOA) [30,31]. Because low muscle mass is associated with greater functional impairments [32] and preoperative functional status predicts the functional recovery pattern [33,34,35], potential sarcopenia in such obese older people with KOA who are undergoing TKR may have additive impacts on postoperative outcomes. In addition, post-surgery muscle attenuation commonly occurs by decreased muscle mass [36] and muscle volume [37,38,39,40,41] from early weeks up to two years after total joint replacement in legs. Moreover, obesity is associated with muscle attenuation. Therefore, obese older adults may suffer potential risks of sarcopenia, especially those who are undergoing TKR. Sarcopenia may occur in end-stage KOA and persist even after TKR, especially for those who are obese, since the prevalence of sarcopenic obesity ranges from 3% to 35% in KOA population, dependent on the diagnostic criteria for classification of sarcopenia and obesity [27,42]. However, whether sarcopenic obesity interferes with surgical outcomes remains unclear. Identifying the role of sarcopenic obesity in recovery after TKR might facilitate the optimization of treatment and identify patients with a poor outcome risk.

Malnutrition is a high risk factor for physical frailty for elder individuals who have KOA [43]. Especially, preoperative nutritional status is significantly associated with surgery complications after TKR [44,45,46]. Therefore, preoperative undernutrition may affect recovery from TKR in elder people with KOA. Whether preoperative undernutrition has any influence on the associations between sarcopenic obesity and ROM recovery needs to be further investigated.

The purpose of this study was to identify the effects of sarcopenic obesity on the knee flexion ROM outcome after TKR in older adults with KOA. We also identified potential confounding factors which may affect the associations between sarcopenic obesity and ROM restoration after TKR. The study hypothesis was that obese patients with or without low muscle mass exhibited higher risks in poor surgical outcomes, identified based on knee flexion ROM after TKR, compared to non-obese patients.

## 2. Materials and Methods

### 2.1. Study Design

This study was conducted based on a retrospective cohort design, used to evaluate the effects of high body mass index (BMI) on post-TKR outcomes [47]. This design adheres to the Strengthening the Reporting of Observational Studies in Epidemiology guidelines [48]. The Institutional Review Board of Taipei Medical University approved our analysis of the patient data contained in the database (Trial number: N202107102). All data used in our study were collected from a rehabilitation center database. Two research assistants, who were trained for standardization of study procedure, collected and extracted the data from the database; in addition, all of the extracted data were further confirmed by one author (C-DL). Patient characteristics and demographics data including age, BMI, comorbidity, nutrition status, side of TKR surgical leg, Kellgren and Lawrence (K-L) grade of the surgical leg, surgical time, length of inpatient stay (LOS) after surgery (in day), and outpatient follow-up time period (in week) were extracted and analyzed. The patients’ global comorbidities were evaluated using Cumulative Illness Rating Scale, which assesses the comorbidity status of older individuals [49].

### 2.2. Participants

**Figure 1:**
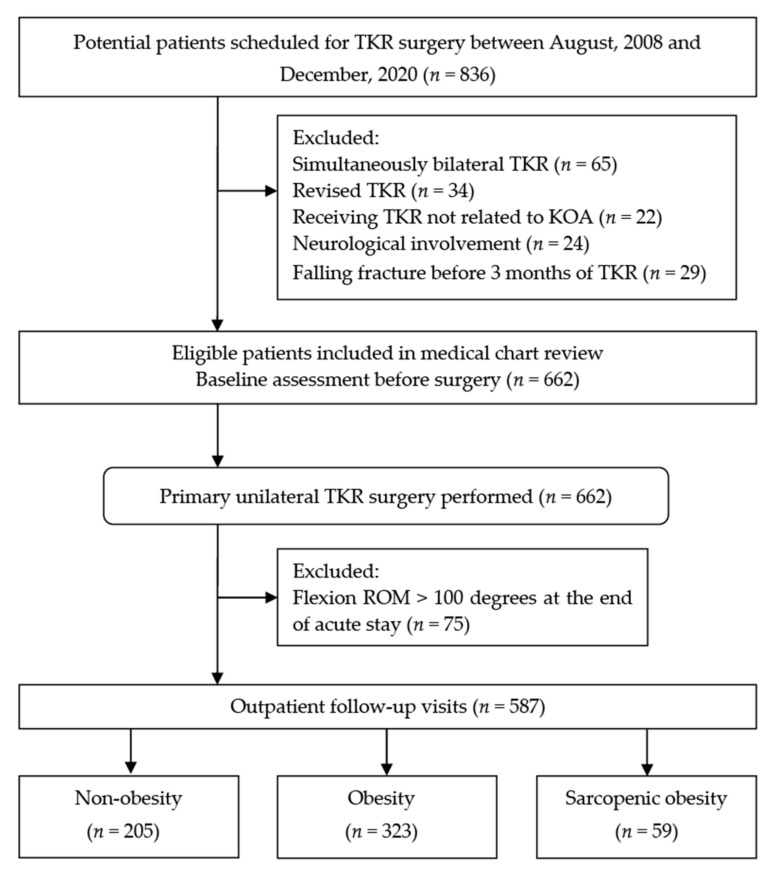
Flow of patient enrollment and allocation throughout the present study. TKR, total knee replacement; KOA, knee osteoarthritis; ROM, range of motion.

The records of patients who were aged 60 years or older, were diagnosed with end-stage KOA (K-L grade ≥3), and had undergone primary unilateral TKR between August 2008 and December 2020, were reviewed. Enrollment criteria were designed to exclude subjects from entry who (1) had comorbidity which was not regularly controlled by medicine including insulin-dependent diabetes, hypertension, and hepatic disease. Such uncontrolled comorbidities may have impacts on an individual’s physiologic reserve [50], especially those who are undergoing an acute event (e.q. TKR surgery) [51]; (2) a history of internal cancer requiring surgery, neurological diseases, or falling fracture in legs before six months of surgery; (3) underwent simultaneously bilateral or a revision TKR; (4) patients who had a knee flexion ROM < 125 degrees at inpatient discharge; the details were described in the following (Section 2.6). A flowchart depicting the patient selection process and study group assignment is illustrated in Figure 1.

### 2.3. Assessment of Nutritional Status and Risk of Undernutrition

Preoperative nutritional status was assessed using Mini Nutritional Assessment Short Form scale (MNA-SF) [52,53]. The MNA-SF significantly predicts survival outcome for Asian hospitalized geriatric patients [54] and has good validity for screening malnutrition in elder populations [55,56]. Two senior research assistants, who had been trained by a nutritionist with a two-week nutritional course, collected the data on the inpatient admission (before surgery) and performed the assessment of nutritional status for each patient.

The total sum scores for the MNA-SF range from 0 to 14 points, with 14 points indicating the best nutritional state possible [52]. An MNA-SF score of ≤7, 8–11, and ≥12 points indicates malnutrition, possible malnutrition, and well-nourished, respectively. In the present study, the patients who obtained a total score of MNA-SF < 12 points were identified as having potential risk of undernutrition [53].

### 2.4. Identification of Sarcopenic Obesity

A BMI cutoff of value 25.0 kg/m^2^, identified by the World Health Organization for Asian population, was used to define obesity in this study cohort [57]. Low muscle mass was identified based on appendicular lean mass (ALM) measurements (in kg), normalized for height to provide AMI [= ALM/height^2^ (in kg/m^2^)]. We followed Wen’s method using an established equation model to estimate ALM [58], which has been validated and used to assess muscle mass for older Chinese population [59,60]. The ALM equation model was established based on anthropometric measurements as follows:

ALM = 0.193 × body weight (kg) + 0.107 × height (m) − 4.157 × gender (male = 1, female = 2) − 0.037 × age (year) − 2.631

Using dual-energy X-ray absorptiometry as the standard reference, the adjusted *R*^2^ of the ALM equation model was 0.90 with a standard error of 1.63 kg [58]. A cutoff value of ALM to define low muscle mass in Asians were based on the diagnostic criteria established by the Asian Working Group for Sarcopenia [61]. Patients were classified as having low muscle mass if the ALM <7.0 kg/m^2^ in men and <5.4 kg/m^2^ in women.

### 2.5. Measurement and Follow up of Knee Flexion Range of Motion

Active knee flexion ROM was measured by two orthopedic doctors before surgery (baseline), at the end of inpatient stay, and at outpatient follow-up admissions. After inpatient discharge, each patient was asked to admit outpatient follow-up clinic one to two times per month until a full recovery of knee flexion ROM (i.e., 130 to 135 degrees [62,63]) was achieved. In addition, the outpatient follow up for each patient was continued for a time period as long as nine months to ensure the maximum gains in knee ROM after TKR, despite recovery of knee flexion ROM reaching a peak at 3−6 months after TKR [13,62].

Active knee flexion ROM was measured using a long-arm goniometer. Patients were asked to lie in supine position and actively bend the involved knee to the maximum extent. Reproducibility of goniometric measurement of active knee flexion is acceptable with an intraclass correlation coefficient of 0.89 for patients receiving TKR [64].

### 2.6. Identification of Poor Surgical Outcome

Poor surgical outcome was identified based on knee flexion ROM, which is an indicator for clinicians to determine the success of TKR surgery [9,10,11,12,13]. In the present study, a cut-off value of 125 degrees of knee flexion was used for poor outcome following TKR based on the following reasons: (1) a knee flexion of 125 degrees permits older individuals to perform the majority of activities in daily life [65,66,67]; (2) a knee flexion ROM < 125 degrees leads to function limitations after TKR [21]; and (3) those who have a knee flexion ROM < 125 degrees tend to experience lower walking capability after TKR [68]. At the end of nine-month outpatient follow-up period, which is defined above, the patients who exhibited a knee flexion ROM < 125 degrees were classified as suffering poor outcome after TKR surgery. The present analysis was limited to patients who had a knee flexion ROM < 125 degrees at inpatient discharge, indicating that all patients were functioning at a low level of ROM at the beginning of outpatient follow-up.

In addition, for each patient, the time period (in week) required to yield a knee flexion ROM ≥ 125 degrees from inpatient discharge was estimated on the basis of the outpatient follow-up data. As mentioned above, all data were collected, assessed, and calculated by the two trained research assistants and the author (C-DL) confirmed all of the extracted data.

### 2.7. Statistical Analysis

One-way ANOVA was used to analyze continuous variables and chi-squared analysis was employed for categorical variables. Post-hoc analysis was performed using the Bonferroni method where the equal variance was assumed. If the homogeneity of group variance was not assumed, the Games-Howell test was performed.

Kaplan-Meier curve analysis was performed to measure the probability of poor postoperative outcome using time period for yielding a knee flexion ROM ≥ 125 degrees as the underlying time scale. In the Kaplan-Meier curve analysis, patients were divided into subgroups by body composition. The log-rank test was used to compare the probabilities of poor surgical outcome among body composition subgroups.

For estimating the associations between different obese groups and ROM restoration after TKR, Cox regression models were established to calculate the hazard ratios (HRs) and the corresponding 95% confidence intervals (CIs) of postoperative poor outcome, with reference to nonobese group and using potential confounders as covariates. The confounding factors which may affect post-TKR outcomes included patients’ age [69,70,71,72], sex [70,72,73,74], comorbidity [75,76], preoperative ROM of knee flexion [69,72,74,77,78], LOS [79], preoperative nutritional status [80,81,82], and outpatient follow-up duration.

A statistician who was blinded to the group allocation was consulted regarding the statistical approach in this study. All differences with *p* < 0.05 were defined as statistically significant. SPSS Statistics (version 22.0; IBM, Armonk, NY, USA) was used for all analyses.

## 3. Results

### 3.1. Patient Demographics and Clinical Characteristics

After 174 patients who did not match the inclusion criteria were excluded, 662 eligible patients who underwent a primary unilateral TKR were included in the medical chart review. Finally, a total of 587 patients (144 men, 443 women) were included for follow up and analyses whereas 69 patients who exhibited a knee flexion < 125 degrees at inpatient discharge were excluded from further investigations. Based on the BMI and AMI cutoff values for obesity and low muscle mass in Asian individuals, respectively, all included patients were stratified into to three body-composition groups: non-obesity (*n* = 205), obesity without low muscle mass (*n* = 323), and obese with low muscle mass (*n* = 59).

Demographic characteristics of the included patients are listed in Table 1. The sarcopenic-obese patients aged nearly 4 years older than those in the non-obese group (*p* < 0.001), whereas obese patients were younger compared with the non-obese peers (mean = 67.8 versus 70.9 years). Between the sarcopenic obesity and non-obesity groups, the mean values of comorbidity score (10.9 versus 8.4 points), number of patients who were at risk of undernutrition (27/59 versus 76/205), preoperative (101 versus 118 degrees) and inpatient-discharge (94 versus 102 degrees) knee flexion ROMs, LOS (6.2 versus 5.3 days), and outpatient follow-up duration (19.5 versus 13.7 weeks) differed significantly (all *p* < 0.01); similar results were observed between the obesity and non-obesity groups. No significant intergroup difference in proportion rates of women, surgical time, side of surgery leg, or K-L grade of the involved leg was observed (all *p* > 0.01).

### 3.2. Survival Time for ROM Recovery after TKR

A total of 378 (64.4%) patients in the study cohort recovered a knee flexion ROM > 125 degrees during an overall follow-up duration of 15.8 weeks (range 1–36 weeks), among which 159 (77.6%) in the non-obesity, 194 (60.1%) in the obesity, and 25 (42.4%) in the sarcopenic-obesity group.

**Figure 2:**
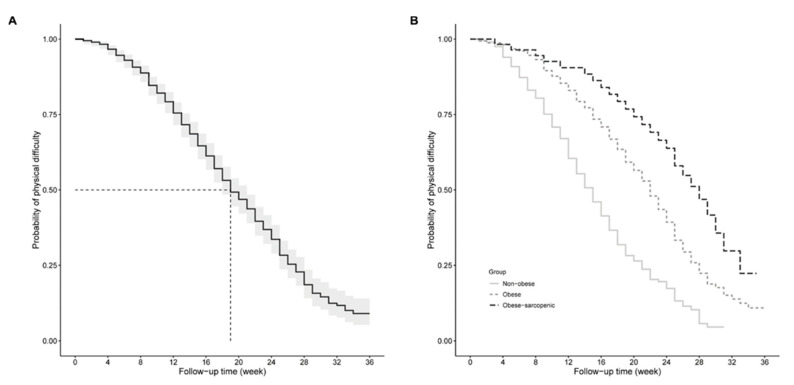
Kaplan-Meier survival curve for time to yield poor surgery outcome by (**A**) overall study cohort and (**B**) body composition type. Time scale on X-axis represents the time point exceeding a knee range of motion of 125 degrees after inpatient discharge.

Kaplan-Meier analysis showed an overall median time of 19 weeks for all patients in the study to yield successful recovery in knee flexion ROM after TKR (Figure 2A). Patients in the obesity and sarcopenic obesity groups had a higher probability of poor surgery outcome (i.e., ROM < 125 degrees) over the postoperative follow-up time period compared to their non-obese peers (all *p* < 0.001; Figure 2B). In addition, those who were classified as having sarcopenic obesity experienced a higher probability of poor surgery outcome compared with purely obese patients (*p* < 0.01). The median survival time of poor surgery outcome for non-obese patients was 15 weeks whereas the obesity group (median survival time = 22 weeks) as well as the sarcopenic obesity group (median survival time = 28 weeks) took relatively longer time periods to recover a knee flexion ROM ≥ 125 degrees (Figure 2B).

### 3.3. Associations of Sarcopenic Obesity with ROM Restoration following TKR

Table 2 shows the results of Cox regression analyses determining the effect of different body-composition types on poor ROM recovery. The crude HR of 3.63 (95% CI, 2.37−5.56) indicates a risk of poor ROM recovery that was 3.6 times higher in sarcopenic-obese patients compared with the referent peers who were neither sarcopenic nor obese preoperatively. Controlling for age, sex, comorbidity score, risk of undernutrition, preoperative ROM, LOS, and outpatient follow-up duration, the HR of poor ROM recovery in sarcopenic obesity group reduced to 1.68 (95% CI, 1.06−2.66) with reference to non-obese group; similar results were observed in the obesity group (adjusted HR = 1.35, *p* < 0.05).

## 4. Discussion

### 4.1. Summary of the Main Findings

This study determined the effects of sarcopenic obesity and its confounding factors on surgery outcome in terms of ROM restoration for individuals with KOA. The results indicated that patients with sarcopenic obesity, as well as those with pure obesity, suffered a higher probability of poor ROM recovery over a follow-up time period of 34 weeks after TKR, compared with the non-obese peers. In addition, the sarcopenic obesity group was at greater risk of suffering poor ROM recovery with an HR 1.6 times greater than the reference group, adjusted for age, sex, comorbidity, risk of undernutrition, LOS, preoperative ROM, and outpatient follow-up duration.

### 4.2. Demographics and Characteristics in the Study Cohort

Demographic results in this study showed that patients were older in the sarcopenic obesity group (mean age = 75.1 years) compared with the non-obese group (mean age = 70.9 years). In addition, the proportions of patients who were aged ≥ 70 years within the sarcopenic obesity group (49/59, 83%) were higher than that within the non-obesity group (161/205, 46%). Our results are supported by previous studies reporting dramatic muscle mass loss occurring at ≥70 years of age [83,84]. In addition, obese patients in this study tended to be significantly younger than the non-obese patients. Similar results concerning effects of obesity on surgery outcome following TKR have been reported in prior literatures [7,8,85].

Another finding of particular interest was that patients in both obesity and sarcopenic obesity groups experienced an inpatient stay approximately one day longer than the non-obese peers did. Our results were consistent with previous studies demonstrating that obese patients (BMI ≥ 30 kg/m^2^) expended a significantly longer time period of hospital stay than those with BMIs < 30 kg/m^2^ did (*p* < 0.001) after total knee or hip arthroplasty [7,85,86,87]. Additionally, a higher comorbidity score along with a longer period of inpatient stay was noted in our obese patients compared with the referent peers and such findings were consistent with prior results [7,85]. Such obese patients who have higher BMIs along with higher comorbidity scores are expected to experience a longer period of hospital stay since BMI and comorbidities predict length of hospital stay after TKR [87,88].

### 4.3. Prevalence of Sarcopenic Obesity and Nutritional Status in End-Stage Knee Osteoarthritis

Sarcopenic obesity is closely associated with radiographic KOA [31], and has become a phenotype in the elder population with lower-limb osteoarthritis [23,89]. Low muscle mass as well as high body fat become evident with disease progression of KOA [24,90], sarcopenic obesity becomes prevalent at late stage of KOA. The overall sarcopenic obesity prevalence at end-stage KOA has been estimated to be 2.7−17.2% for the elder population in Asia [26,31,91] and 2.6−9.4% in other areas worldwide [27,32], regardless of sex, age, and identification criteria of sarcopenic obesity. In this study cohort, 59 out of 587 (10.1%) patients with moderate or severe KOA were classified as having sarcopenic obesity, which agrees with previous results.

In the present study, 195 out of 587 patients (33.2%) had an MNA-SF score < 12, indicating high risk of undernutrition. Our findings were in agreement with prior results reporting 32.4% of older individuals with KOA are at high risk for malnutrition assessed by MNA-SF [43]. We further identified that the proportion of patients at high risk of undernutrition was greater in the sarcopenic obesity (45.8%) compared with non-obese control (37.1%). Our findings are supported by previous results indicating that the prevalence of patients at high risk of malnutrition (47%) is greater among frail individuals with KOA compared with the non-frail peers (19%) [43]. Based on our results, sarcopenic obesity was more likely to experience undernutrition than non-obese peers did in KOA population.

### 4.4. Obesity and Range of Motion in Knee Osteoarthritis

Preoperative ROM has been identified as the principal predictor of eventual knee flexion after TKR [69,72,92]. In the present study, significant differences among the body-composition groups were noted in knee flexion ROM before surgery. After adjustment for such potential confounding variables, our patients in the obesity and sarcopenic obesity groups exhibited higher probabilities of poor ROM recovery compared with their non-obese peers. Our results may indicate that obesity exerted negative effects on restoration of ROM after TKR. The underlying mechanism through which obesity affects knee flexion ROM in older individuals with KOA remains unclear. The following conclusions of previous studies may support our findings: first, our results were in agreement with prior literatures indicating that obesity is associated with poorer postoperative knee flexion ROM [93,94,95,96]; secondly, given the fact that earlier calf-thigh impingement of the thigh and lower leg results from increased posterior soft tissue girth behind the knee [95,97], it is rational that obese patients experience reduced postoperative ROM.

### 4.5. Sarcopenic Obesity and Its Risk of Poor Recovery in Range of Motion after Total Knee Replacement

Recovery in knee ROM following TKR is likely to be negatively impacted by either obesity [93,96] or sarcopenia [98]. In agreement with prior findings, survival analysis results in the current study demonstrated that the obesity group took a time period of 7 weeks longer than the non-obese control did to recover a knee flexion over 125 degrees. We further identified that the sarcopenic obesity group took a longer time period to achieve knee flexion ≥ 125 degrees after TKR than the obesity and non-obesity group did. Accordingly, sarcopenic-obese patients were determined to have the highest probability of poor surgery outcome (i.e., flexion ROM < 125 degrees) among the body-composition groups. Our results may indicate that obesity alone exerted negative impacts on surgery outcome after TKR whereas sarcopenic obesity had greater negative effects on post-TKR function recovery than obesity alone did, particularly the restoration of knee flexion ROM.

Cox regression analyses results in this study showed that sarcopenic-obese patients as well as purely obese patients suffered high risks of poor ROM recovery post-surgery, with reference to the non-obese peers. In addition, sarcopenic-obese patients were likely to yield a relatively higher risk of post-TKR poor ROM recovery compared with the obese peers without sarcopenia. Our findings in obesity group were supported by previous studies concerning associations between high body fat (or BMI) and ROM restoration after TKR [7,93,94,95,96]. However, few of the prior authors discussed the associations between sarcopenic obesity and surgery outcome following TKR. According to our results, sarcopenic obesity appears to exert impacts on function recovery, particularly the restoration of knee flexion ROM, independent to age, sex, comorbidity, malnutrition risk, LOS, preoperative flexion ROM, and the outpatient follow-up period. Some reasons may explain our findings as follows: first, knee ROM at inpatient discharge has been identified as a relevant indicator for knee flexion outcome after TKR [74]; in addition, insufficient gains in knee flexion ROM at early period after TKR predict its poor recovery at later follow up [9]. Therefore, our sarcopenic-obese patients who exhibited a minor ROM may take a longer period to regain ROM and obtain minor knee flexion at a long-term follow up, compared to the non-obese patients.

### 4.6. Study Limitations 

A number of certain limitations to this study need to be highlighted. First, our study included only older individuals with moderate to severe KOA; therefore, the results cannot be generalized to those who have mild KOA (K-L grade < 3). In addition, our results might not be generalizable to all TKR types. Further investigation is necessary regarding whether sarcopenic obesity affects function outcomes post-surgery in patients with other preoperative diagnoses, such as rheumatoid arthritis, are similar to those for KOA. Secondly, the needs or prescriptions for outpatient rehabilitation were not assessed. Because of this, the rehabilitation exercise program following TKR such as stretch or ROM exercises may have positive effects on recovery rate of knee flexion ROM, the time duration required to exceed a knee ROM ≥ 125 degrees may be underestimated in our analyses. Further studies are needed to assess whether outpatient rehabilitation, including exercise training types and durations, has any effect on the associations between sarcopenic obesity and ROM restorations following TKR. Thirdly, medication prescribed for pain control also represents a potential confounding factor. We did not consider drug use for pain in our analysis of the results of the ROM measure. Future studies on whether pain medications have a significant intragroup contribution to the recovery rate of knee ROM after TKR are warranted. Fourth, the assessment of nutritional status using MNA-SF may underdiagnose malnutrition for the patients enrolled in this study [99,100]. Fifth, we did not conduct a blinding process performed either at the data collection or data interpretation stage despite all of the collected and extracted data being further confirmed by one study author. Results in the current study should be cautiously interpreted due to potential detection bias. Finally, potential confounders, such as disease duration [101,102], may have made contributions when measuring acute outcomes after TKR. However, disease duration was not assessed or included as covariates for analysis in this study; therefore, differences in risk of poor ROM outcome among the body-composition groups may had been overestimated.

## 5. Conclusions

This study demonstrated that obesity, as well as sarcopenic obesity, independently had negative effects on surgery outcome following TKR in terms of yielding high risks of poor recovery in knee flexion ROM. Sarcopenic obesity appears to have greater risk of poor ROM recovery after TKR compared with obesity alone for older individuals who had moderate to severe KOA. The results of the current study have implications for clinicians setting rehabilitation strategies to optimize ROM restorations after TKR, especially for those who are identified as sarcopenic obesity pre-surgery.

## Figures and Tables

**Table 1:** Demographic characteristics of participants.

Items	Non-Obese			Obese			Obese-Sarcopenic			*p* Value
	mean	±	SD	mean	±	SD	mean	±	SD	
*n*	205			323			59			
Women, *n* (%)	160	(78.0)		249	(77.1)		39	(66.1)		0.15 ^d^
Age (years)	70.9	±	6.9	67.8	±	6.3^a^	75.1	±	6.1 ^a^	<0.001 ^b^
Age category, *n* (%)										<0.001 ^d^
<65 years	44	(21.5)		156	(48.3)		10	(16.9)		
70−74.9 years	101	(17.1)		105	(32.5)		21	(35.6)		
≥75 years	60	(29.2)		62	(19.2)		28	(47.5)		
BMI (kg/m^2^)	22.9	±	1.8	30.6	±	3.9 ^a^	27.0	±	2.1 ^a^	<0.001 ^c^
ALM (kg)	16.0	±	3.2	17.9	±	3.6 ^a^	13.8	±	2.8 ^a^	<0.001 ^c^
AMI (kg/m^2^)	6.64	±	0.91	7.56	±	0.98 ^a^	5.96	±	0.75 ^a^	<0.001 ^c^
CIRS score	8.4	±	5.7	10.1	±	5.5 ^a^	10.9	±	6.2 ^a^	0.001 ^c^
Surgical time (min)	149	±	35	152	±	39	155	±	33	0.35 ^b^
LOS (day)	5.3	±	1.6	6.5	±	2.4 ^a^	6.2	±	1.9 ^a^	<0.001 ^c^
Follow-up time (week)	13.7	±	7.3	16.4	±	7.7 ^a^	19.5	±	8.9 ^a^	<0.001 ^c^
TKR leg, Right, *n* (%)	123	(60.0)		181	(56.0)		31	(52.5)		0.51 ^d^
KL grade, TKR leg, *n* (%)										0.33 ^d^
3	103	(50.2)		165	(51.1)		24	(40.7)		
4	102	(49.8)		158	(48.9)		35	(59.3)		
MNA-SF <12 points	76	(37.1)		92	(28.5)		27	(45.8)		0.01 ^d^
Number of comorbidities, *n* (%)										
Hypertension	129	(62.9)		228	(70.6)		40	(67.8)		0.19 ^d^
DM	37	(18.0)		139	(43.0)		22	(37.3)		<0.001 ^d^
Cardiopulmonary disease	94	(45.9)		174	(53.9)		29	(49.2)		0.19 ^d^
Leg fracture	12	(5.9)		15	(4.6)		3	(5.1)		0.83 ^d^
Knee flexion ROM (degree)										
Presurgery	118	±	14	108	±	13 ^a^	101	±	15 ^a^	<0.001 ^b^
Inpatient discharge	102	±	10	95	±	10 ^a^	94	±	10 ^a^	<0.001 ^b^

^a^ A significant difference compared with the reference group, *p* < 0.05. ^b^ One-way analysis of variance. Post-hoc analysis was performed using the Bonferroni test. ^c^ One-way analysis of variance. Post-hoc analysis was performed using the Games-Howell test. ^d^ Chi-Square Test. AMI, appendicular mass index; BMI, body mass index; CIRS, Cumulative Illness Rating Scale; DM, diabetes mellitus; ROM, gait speed; K-L grade, Kellgren-Lawrence grade; MNA-SF, Mini Nutritional Assessment Short Form scale; SD, standard deviation; TKR, total knee replacement.

**Table 2:** Hazard ratios for proportional hazards models evaluating the associations of body-composition types with poor recovery in knee flexion.

Body-Composition Group	Crude Model			Adjusted Model ^a^		
	HR	(95% CI)	*p*	HR	(95% CI)	*p*
Non-obesity	Reference			Reference		
Obesity	2.10	(1.68, 2.62)	<0.001	1.35	(1.04, 1.75)	0.02
Sarcopenic obesity	3.63	(2.37, 5.56)	<0.001	1.68	(1.06, 2.66)	0.03

^a^ Model was adjusted using age, sex (coded as men = 1, sex = 2), comorbidity score, risk of undernutrition (coded as yes = 1, no = 0), preoperative range of motion, length of inpatient stay, and outpatient follow-up time as covariates. HR, hazard ratio; 95% CI, 95% confidence interval.

## Data Availability

The data presented in this study are available on request from the corresponding author. Public data sharing is not applicable to this article due to ethical considerations and privacy restrictions.
